# Functional identification of islet cell types by electrophysiological fingerprinting

**DOI:** 10.1098/rsif.2016.0999

**Published:** 2017-03-08

**Authors:** Linford J. B. Briant, Quan Zhang, Elisa Vergari, Joely A. Kellard, Blanca Rodriguez, Frances M. Ashcroft, Patrik Rorsman

**Affiliations:** 1 Oxford Centre for Diabetes, Endocrinology, and Metabolism, Radcliffe Department of Medicine, University of Oxford, Churchill Hospital, Oxford OX3 7LE, UK; 2 Department of Computer Science, University of Oxford, Oxford OX1 3QD, UK; 3 Department of Physiology, Anatomy, and Genetics, University of Oxford, South Parks Road, Oxford OX1 3PT, UK; 4 Metabolic Research, Department of Physiology, Institute of Neuroscience and Physiology, University of Göteborg, SE-405 30 Göteborg, Sweden

**Keywords:** islet electrophysiology, logistic regression, α-cell, β-cell, δ-cell, conductance-based models

## Abstract

The α-, β- and δ-cells of the pancreatic islet exhibit different electrophysiological features. We used a large dataset of whole-cell patch-clamp recordings from cells in intact mouse islets (*N* = 288 recordings) to investigate whether it is possible to reliably identify cell type (α, β or δ) based on their electrophysiological characteristics. We quantified 15 electrophysiological variables in each recorded cell. Individually, none of the variables could reliably distinguish the cell types. We therefore constructed a logistic regression model that included all quantified variables, to determine whether they could together identify cell type. The model identified cell type with 94% accuracy. This model was applied to a dataset of cells recorded from hyperglycaemic βV59M mice; it correctly identified cell type in all cells and was able to distinguish cells that co-expressed insulin and glucagon. Based on this revised functional identification, we were able to improve conductance-based models of the electrical activity in α-cells and generate a model of δ-cell electrical activity. These new models could faithfully emulate α- and δ-cell electrical activity recorded experimentally.

## 1. Introduction

The pancreatic islet is composed of three main cell types: α-, β- and δ-cells [1,2]. All three cell types are electrically excitable and use electrical signals to regulate hormone release [3–5]. These hormones—glucagon, insulin and somatostatin, respectively—all have a role in normalizing plasma glucose [6–8]. In type 2 diabetes mellitus (T2DM), both glucagon and insulin secretion are impaired [9,10]. This impairment has been linked to changes in the electrical properties of α- and β-cells [11–14]. Determining the mechanisms by which islet cells couple electrical activity to hormone secretion is therefore fundamental for understanding normal glucose homeostasis and the pathophysiology of T2DM.

The whole-cell patch-clamp technique, applied to intact islets, is the perfect experimental paradigm for understanding the electrophysiological properties of islet cells. However, within a mouse islet, the different cell types are not present in equal proportions; β-cells are the most abundant (70–80% of all cells), with α-cells (15–20%) and δ-cells (5–10%) being relatively sparse [15]. Thus, whereas there have been great advances in our understanding of the electrical properties of β-cells and how they couple to insulin secretion in both health and disease [16–18], progress has been slower and shrouded in controversy for α-cells [4,19–21]. For δ-cells, there remains great uncertainty, even with regard to fundamental aspects of the metabolic regulation of their electrical activity.

This has motivated the development of strategies to improve identification of islet cell type. β-cells can be separated from non-β-cells by autofluorescence-activated cell sorting [22]. Although this can purify β-cells and α-cells by 80–90%, it has the drawback of removing cells from their paracrine environment—an environment necessary for maintaining normal electrophysiological and secretory function [8,23–27]. Recent efforts have been made to produce fluorescent labels for particular islet cell types in the mouse [28–30]. However, it is not straightforward to distinguish labelled and non-labelled cells in the intact islet owing to fluorescence emission from cells deeper in the tissue layer. For this reason, islet cells from such transgenic mice are often dispersed into single cells [29,31–33]. This allows labelled cells to be identified, but again removes them from their paracrine environment. Many experiments are therefore still performed on intact islets harvested from normal (i.e. not genetically modified) mice, where cell type must be distinguished by reference to established differences in the electrophysiological properties of α-, β- and δ-cells or post-recording, using immunocytochemistry. Here, we explored whether the electrophysiological properties can be used to reliably ‘functionally identify’ each cell type.

Currently, electrophysiological identification of cell type (α, β or δ) relies on two criteria. The first is that β-cells are larger than non-β-cells [3,34–44]. The second is that α-, β- and δ-cells possess distinct ionic channels or similar channels that exhibit different properties. For example, β-cells exhibit non-inactivating K^+^ currents and a voltage-gated Na^+^ current that inactivates at very hyperpolarized potentials [37,39,45]. In contrast, there is evidence that non-β-cells express an A-type transient K^+^ current [29,31,38,46–48], a Na^+^ current with depolarized inactivation properties relative to the β-cell [11,29,39–41,44–47,49,50] and T-type Ca^2+^ channels [3,38,46]. Several laboratories have used these different electrophysiological fingerprints to distinguish between α-, β- and δ-cells [11,13,31,34–36,39–44,46–48,50–53].

Here we reviewed the electrophysiological fingerprints of mouse α-, β- and δ-cells. We recorded and analysed a large dataset of whole-cell voltage-clamp recordings (288 recordings) made from cells in intact mouse islets, whose cell type was subsequently unequivocally determined by immunocytochemistry. We used these data to investigate the validity of these properties for cell identification and to produce a mathematical model for identifying islet cell type. We show that this model can reliably identify islet cell type and can be successfully used to monitor transdifferentiation of cells in a diabetic mouse model (βV59M) [54]. Our findings demonstrate that the electrophysiological properties of α- and δ-cells differ somewhat from what has previously been deduced. We finally used this amended information to improve reported conductance-based models of the electrical activity in α-cells and δ-cells and show that these revised models faithfully resemble experimentally recorded action potential shape.

## 2. Methods

### 2.1. Animals used in this study

Recordings from 288 cells in islets from five different strains of mouse with a normoglycaemic phenotype were used in this study. The mouse strains were NMRI, C57BL/6, EPAC2-KO [55], GYY [32] and SST-Cherry [56]. Islets from a mouse model with a hyperglycaemic phenotype were also used, together with littermate controls [54]. These mice have a valine-to-methionine substitution in the Kir6.2 subunit of the ATP-sensitive K^+^ (*K*_ATP_) channel in β-cells (βV59M mice). This dataset consisted of 13 recordings from βV59M mice, and 15 from littermate controls.

### 2.2. Preparation of pancreatic islets

Mice were killed by cervical dislocation, and islets isolated by liberase digestion (schedule 1 procedure). Islets were used for acute experiments and were not maintained in tissue culture for less than 16 h. A new islet was used for each cell recording.

### 2.3. Whole-cell patch-clamp recordings

Whole-cell currents were recorded in intact islets using the standard whole-cell configuration. Measurements were performed using an EPC-10 patch-clamp amplifier and Pulse software (HEKA Electronics, Lambrecht/Pfalz, Germany). Currents were filtered at 2.9 kHz and digitized at more than 10 kHz. Currents were compensated for capacitive transients and leak current subtraction was conducted. The extracellular medium consisted of (mM) 118 NaCl, 20 tetraethylammonium-Cl (TEA-Cl), 5.6 KCl, 1.2 MgCl_2_, 5 HEPES (pH 7.4 with NaOH), 2.6 CaCl_2_ and 1 d-glucose. Two intracellular (pipette) solutions were used (solution 1 and solution 2). Solution 1 contained (mM) 125 K-glut, 10 KCl, 10 NaCl, 1 MgCl_2_, 5 HEPES, 3 MgATP and 0.05 EGTA (KOH buffered). Solution 2 contained 15 Cs-glut, 10 CsCl, 10 NaCl, 1 MgCl_2_, 5 HEPES, 3 MgATP, 0.05 EGTA (CsOH buffered). All chemicals were from Sigma-Aldrich. Only recordings with an access resistance of less than 50 MΩ were used for analysis.

### 2.4. Identification of cell type by immunocytochemistry

In all recordings, cell identity (α, β or δ) was subsequently established by immunocytochemistry. Biocytin (0.5 mg ml^−1^) was included in the intracellular solution to allow identification of the cell recorded from. Following voltage-clamp experiments, islets were fixed with 4% formaldehyde in phosphate-buffered saline (PBS) overnight and permeabilized with 0.3% Triton X-100. Non-specific binding was blocked by pre-treatment for 2 h with 5% normal goat serum before incubating with the different primary antibodies for 4–12 h (guinea pig anti-insulin (Abcam, Cambridge, UK), sheep anti-glucagon (Sigma-Aldrich, St Louis, MO) and rabbit anti-somatostatin (Vector Labs, Burlingame, CA)). After washing with PBS, the islet was incubated for 1 h in secondary antibodies (Alexa 633 goat anti-guinea pig (insulin), Alexa 405 goat anti-mouse (glucagon) and Alexa 543 goat anti-rabbit (somatostatin)). Biocytin labelling was visualized by using Alexa Fluor 488 conjugated streptavidin (0.04 mg ml^−1^; Thermo Fisher). Islets were then washed and imaged on a confocal microscope (Axioskop 2 upright microscope fitted with a Zeiss LSM 510 meta confocal and a chameleon multiphoton module).

### 2.5. Electrophysiological variables

For every cell, several electrophysiological variables were recorded and characterized (table 1). All analyses were conducted blinded to cell type. The electrophysiological variables quantified are described in appendix A.

**Table 1.** Variables quantified/characterized and used in the construction of the multinomial logistic regression model of islet cell type.

variable (*X_i_*)	description	continuous/categorical
animal strain	strain of the animal from which cell recording taken	categorical [1 = C57BL6; 2 = EPAC2-KO; 3 = Glu-; 4 = NMRI; 5 = SST-Cherry]
double sigmoid	does the steady-state inactivation of Na^+^ currents exhibit a double or single sigmoidal shape?	categorical [0 = single; 1 = double]
*V*_2 h_	half-inactivation of Na^+^ current	continuous
*k*_h_	slope factor of inactivation of Na^+^ current	continuous
*R*^2^	goodness-of-fit of the sigmoidal function to steady-state Na^+^ current data	continuous
*I*_max_	maximum Na^+^ current evoked	continuous
*I*_max70_	Na^+^ current evoked from a holding potential of −70 mV	continuous
*C*_cell_	cell capacitance	continuous
*R*_access_	access (series) resistance of recording	continuous
tail current	presence of a tail current in current–voltage data (see §3.5)	categorical [0 = no; 1 = yes]
transient current	presence of a transient outward current (see §3.6)	categorical [0 = no; 1 = yes]
*I*_leak_	leak current of the recording	continuous
*R*_input_	input (seal) resistance of the recording in 1 mM glucose	continuous
ratio current	ratio of *I*_max_ and *I*_max70_	continuous
intracellular solution	solution 1 (K-glut) or solution 2 (Cs-glut)	categorical [solution 1; solution 2]

### 2.6. Multinomial logistic regression model for predicting islet cell type

A multinomial logistic regression model was constructed. For a given set of electrophysiological measures from a specific cell recording, this model can be used to predict the cell type. The model process requires a dataset for constructing (model construction dataset; *N* = 175 cell recordings) and validating (model validation dataset; *N* = 113 cell recordings) the model. A description of this model and the modelling process is given in appendix B.

The multinomial logistic regression model was constructed in SPSS (IBM, Armonk, NY). The model developed was coded into a freely available Matlab toolbox for predicting cell type. The toolbox and SPSS files are available from GitHub (https://github.com/IsletCellType/IsletCellType_GitHub). The toolbox uses the multinomial logistic regression model presented to predict cell type, given a set of user-defined inputs (electrophysiological variables from the recorded cell). We have also made available on GitHub the entire dataset of 288 cell recordings that can be tested with the multinomial regression model.

### 2.7. Statistical tests of electrophysiological variables and analysis

All data are reported as mean ± s.e.m., unless otherwise stated. SD refers to the standard deviation and *N* refers to the number of cell recordings. Statistical significance was defined as *p* < 0.05.

All recorded variables were compared across cell types using one-way ANOVA (Prism5; GraphPad Software, San Diego, CA). If the data passed normality criteria (D’Agostino's test of normality and Bartlett's test of equal variances), a parametric test was conducted with the appropriate post hoc test (Tukey). If the normality criteria were not met, a Kruskal–Wallis test with Dunn's multiple comparison test was conducted.

Some of the variables used to identify cell type, such as the presence/absence of an outward transient current, are categorical (table 1). A contingency table analysis (Pearson's *χ*^2^) will test whether there is an association between this variable and cell type. For post hoc tests, we adopted the approach described by Sharpe [57]; contingency tables were partitioned into 2 × 2 tables, and a Fisher's exact test was conducted [57].

### 2.8. Conductance-based models

Conductance-based (Hodgkin–Huxley-like) models were used to simulate electrical activity in a model of an α-cell and a δ-cell. All conductance-based models were solved numerically in the software package XPPAUT [58] using the variable step size method CVODE with absolute and relative tolerances of 10^−10^. The models are described in appendix C and can be obtained from GitHub (https://github.com/IsletCellType).

In what follows, it will be clear from the context whether we are referring to either (i) a conductance-based model of α- or δ-cell electrical activity or (ii) a multinomial logistic regression model for predicting islet cell type.

## 3. Results

We analysed the electrophysiological variables of 288 cells in intact islets from mice with a normoglycaemic phenotype.

### 3.1. Cell capacitance is an inadequate identifier of islet cell type

**Figure 1.**
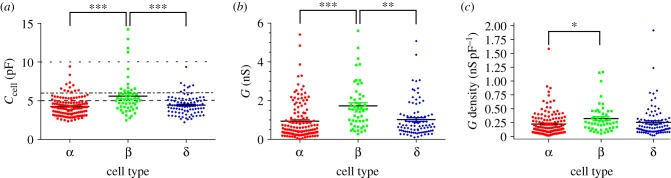
Differences in cell size and resting conductance between islet cell types. (*a*) Cell capacitance (*C*_cell_), (*b*) input conductance (*G*) and (*c*) conductance density (*G*/*C*_cell_) in α-cells (*N* = 141), β-cells (*N* = 56) and δ-cells (*N* = 91). Criteria for identifying cell type based on a cut-off for *C*_cell_, such as >5 pF (dashed; [42]) or >6 pF (dashed-dotted; Andersson *et al.* [34] and Guo *et al.* [13]), are included. One-way ANOVA with Tukey's post hoc test (**p < 0.01; ***p < 0.001). (Online version in colour.)

Cell capacitance (*C*_cell_) in β-cells (5.8 ± 0.3 pF, *N* = 56) was significantly larger than that seen in α-cells (4.2 ± 0.1 pF, *N* = 141; *p* < 0.001) and δ-cells (4.3 ± 0.1 pF, *N* = 91; *p* < 0.001; figure 1*a*). α-Cells and δ-cells did not differ in their cell size (*p* = 0.556). Given that *C*_cell_ is frequently used to identify cell type [13,34,36,39,40,42,44,49], we constructed a multinomial logistic regression model to investigate whether *C*_cell_ alone can identify cell type (equation (B 2) and table 2). The model identified α-cells with 89% accuracy, but poorly identified β-cells (11/40 were identified correctly) and δ-cells (1/62). Thus *C*_cell_ alone is an inadequate indicator of cell type.

**Table 2.** Single electrophysiological variables inadequately identify islet cell type. For each electrophysiological variable, a multinomial logistic regression model (equation (B 2)) was constructed to investigate how accurately this variable can identify cell type on its own. Each row represents a separate model, constructed with one independent variable (*X_i_*). The parameter estimates are relative to the δ-cell reference category. For variable descriptions, see table 1. All models were constructed using the sample values from the model construction dataset (*N* = 175 cells).

variable (*X_i_*)	*B*_0α_		*B*_1α_		*B*_0β_		*B*_1β_		observed/predicted [% correct]			
	mean ± s.e.m.	sig.	mean ± s.e.m.	sig.	mean ± s.e.m.	sig.	mean ± s.e.m.	sig.	*α*	*β*	*δ*	overall
*C*_cell_	0.78 ± 0.31	0.283	−0.12 ± 0.14	0.40	−2.58 ± 0.70	0.00	0.42 ± 0.13	0.002	65/73 [89.0]	11/40 [15.2]	1/62 [6.9]	44.0
*R*_input_	−0.01 ± 0.26	0.97	7.8 ± 9.3×10^−5^	0.40	0.72 ± 0.34	0.03	8.9 ± 2.5×10^−4^	0.00	53/73 [72.6]	23/40 [57.5]	0/62 [0]	43.4
*I*_max_	2.34 ± 0.45	<0.001	3.4 ± 0.6×10^−3^	<0.001	−0.37 ± 0.49	0.45	1.1 ± 0.6×10^−3^	0.07	60/73 [82.2]	0/40 [0]	41/62 [66.1]	57.7
*V*_2 h_	0.49 ± 0.42	0.27	8.0 ± 0.01×10^−3^	0.39	−3.89 ± 0.67	<0.001	−0.06 ± 0.01	<0.001	64/73 [87.7]	31/40 [77.5]	0/62 [0]	54.3
*k*_h_	−0.89 ± 0.42	0.052	0.12 ± 0.05	0.016	−2.6 ± 0.56	<0.001	0.22 ± 0.05	<0.001	42/73 [57.5]	8/40 [20]	36/62 [58.1]	49.1
*I*_leak_	0.70 ± 0.26	<0.001	0.04 ± 0.01	0.007	−0.39 ± 0.28	0.17	0.002 ± 0.01	0.80	61/73 [83.6]	0/40 [0]	23/62 [37.1]	48.0
*R*_access_	0.1 ± 0.5	0.83	2.5 ± 2×10^−3^	0.90	−0.55 ± 0.55	0.34	5.0 ± 2.3×10^−3^	0.83	73/73 [100]	0/40 [0]	0/62 [0]	41.7
double sigmoid	0.20 ± 0.45	0.65	−0.04 ± 0.49	0.93	1.3 ± 0.38	<0.001	−3.16 ± 0.54	<0.001	62/73 [84.9]	32/40 [80]	0/62 [0]	53.7
tail current	−18.8 ± 0.24	<0.001	20.02 ± 0.01	<0.001	−19.4 ± 0.27	<0.001	20.02 ± 0.01	<0.001	73/73 [100]	0/40 [0]	40/62 [64.5]	64.6
transient current	−0.55 ± 0.38	0.15	0.91 ± 0.42	0.03	−18.3 ± 0.22	<0.001	18.2 ± 0.01	<0.001	62/73 [84.9]	0/40 [0]	19/62 [30.6]	47.3

### 3.2. *K*_ATP_ conductance is largest in β-cells

The whole-cell conductance (*G*) was larger in β-cells (1.7 ± 0.2 nS, *N* = 56) than in α-cells (0.9 ± 0.1 nS, *N* = 141; *p* < 0.001) or δ-cells (1.0 ± 0.1 nS, *N* = 91; *p* = 0.005; figure 1*b*). There was no difference in *G* between α-cells and δ-cells (*p* = 0.215).

*G* density (*G* normalized by *C*_cell_) in α-cells (0.22 ± 0.02 nS pF^−1^, *N* = 141) was statistically lower than in β-cells (0.33 ± 0.03 nS pF^−1^, *N* = 56; *p* = 0.017; figure 1*c*). *G* density in δ-cells (0.25 ± 0.03 nS pF^−1^, *N* = 91) was no different from that in β-cells (*p* = 0.184) or α-cells (*p* = 0.536).

### 3.3. Na^+^ currents are largest in δ-cells (not α-cells)

**Figure 2.**
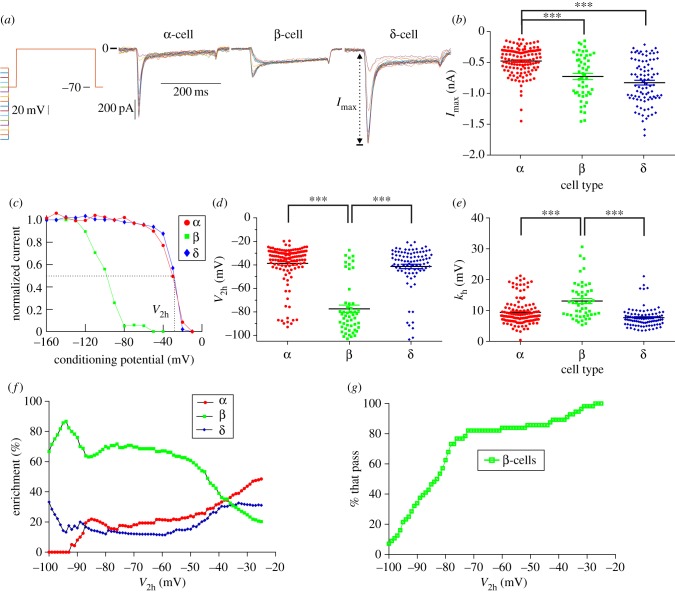
Na^+^ currents are smallest in α-cells. The maximum Na^+^ current evoked (*a*, *I*_max_) by a 200 ms test pulse to 0 mV, following preconditioning pulses, was measured in all cells (*b*). These data were also used to calculate the normalized peak current as a function of the conditioning potential (*c*). For each cell, these data were fitted with a sigmoid to quantify the half-inactivation *V*_2h_ (*d*) and the slope factor *k_h_* (*e*). The number of cells with *V*_2h_ < a fixed cut-off (−25 to −100 mV) was counted. The percentage of these cells that are β-cells (enrichment, *f*) and the percentage of all β-cells that pass this criterion (% that pass, *g*) were then calculated. One-way ANOVA with Tukey's post hoc test (***p < 0.001). *N* = 141 α-cells, *N* = 56 β-cells and *N* = 91 δ-cells. (Online version in colour.)

The maximum amplitude of the Na^+^ current (*I*_max_; figure 2*a*) evoked in α-cells (−465 ± 19 pA, *N* = 141) was significantly smaller than that in β-cells (−720 ± 50 pA, *N* = 56; *p* < 0.001) and δ-cells (−846 ± 37 pA, *N* = 91; *p* < 0.001; figure 2*b*). There was no difference in *I*_max_ between δ- and β-cells (*p* = 0.14). We explored whether *I*_max_ could be used to predict cell type in a multinomial logistic regression model (equation (B 2) and table 2), given that it is frequently used to identify cell type [29,35,39,40,45–47]. The model identified cell type with 57.7% accuracy, and failed to identify any β-cells. Therefore, *I*_max_ alone cannot reliably identify cell type.

### 3.4. *V*_2 h_ cannot reliably distinguish β-cells from non-β-cells

The voltage dependence of steady-state inactivation of the Na^+^ current differed between cell types (figure 2*c–e*). Inactivation in α-cells was half-maximal (*V*_2 h_) at −38.4 ± 1.4 mV (*N* = 141), as observed in pancreatic slices [40]. This value was not statistically different from that in δ-cells (−41.4 ± 1.8 mV, *N* = 91; *p* = 0.187). In contrast, *V*_2 h_ was significantly more hyperpolarized in β-cells (−78.3 ± 3 mV, *N* = 56) than in either α-cells (*p* < 0.001) or δ-cells (*p* < 0.001). There was no difference in *V*_2 h_ between α- and δ-cells (*p* = 0.22).

As it is more hyperpolarized in β-cells, *V*_2 h_ is often used to distinguish β-cells from non-β-cells [11,29,39–41,44–47,49,50]. We therefore explored whether *V*_2 h_ alone could be used to distinguish cell type. We first did this by investigating whether a simple criterion could enrich the β-cell population; the number of cells with *V*_2 h_ < a fixed cut-off were counted. The cut-off ranged from −25 to −100 mV in 1 mV increments. For each cut-off, the numbers of α-, β- and δ-cells that pass this criterion were counted. The percentage of these cells that were β-cells (β-cell enrichment; figure 2*f*) and the percentage of β-cells that pass this criterion (figure 2*g*) were then calculated. As the cut-off became more hyperpolarized, β-cell enrichment increased. However, the percentage of β-cells that passed this criterion also decreased. Therefore, attempting to enrich β-cells with a criterion based on *V*_2 h_ comes with a cost—a drastic decrease in sample size. We further demonstrated that *V*_2 h_ cannot reliably identify cell type by constructing a multinomial logistic regression model of cell type, with one independent variable (*V*_2 h_; equation (B 2)). The model was unable to identify δ-cells (0% correct) and correctly identified cell type with an overall accuracy of 54% only (table 2).

The slope factor of steady-state inactivation was greater in β-cells (*k*_h_ = −13.1 ± 0.8 mV, *N* = 56) than in α-cells (*k*_h_ = −9.5 ± 0.4 mV, *N* = 141; *p* = 0.001) and δ-cells (*k*_h_ = −7.7 ± 0.3 mV, *N* = 91; *p* < 0.001; figure 2*e*). The slope factor was also significantly smaller in α-cells than in δ-cells (*p* < 0.001).

### 3.5. Ca^2+^ tail currents are most prominent in δ-cells

**Figure 3.**
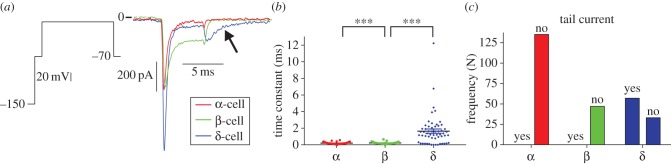
The presence of a slow Ca^2+^ tail current is a feature of δ-cells (not α-cells). (*a*) The presence of a slow tail current was characterized in each of the 288 cell recordings. (*b*) Following a test pulse to 30 mV, a slow tail current was present on the decay to −70 mV if the time constant of decay of the tail current was >1.5 ms. (*c*) The slow tail current was most prevalent in δ-cells. One-way ANOVA with Tukey's post hoc test (***p < 0.001). *N* = 141 α-cells, *N* = 56 β-cells and *N* = 91 δ-cells. (Online version in colour.)

We next analysed slow tail currents in all cells (figure 3*a,b*). The average time constant of decay in δ-cells (1.9 ± 0.2 ms; *N* = 91) was significantly greater than that in α-cells (0.58 ± 0.03, *N* = 141; *p* < 0.001) and β-cells (0.54 ± 0.04, *N* = 56; *p* < 0.001). Slow tail currents were present in 0/141 α-cells, 4/56 (7%) β-cells and 59/91 (65%) δ-cells (figure 3*c*). The presence of a slow tail current in δ-cells was statistically different from that in α-cells (*p* < 0.001) and β-cells (*p* < 0.001). This contrasts with previous studies which have used the presence of a slow tail current to identify α-cells [29,31,38,46].

### 3.6. The presence of a transient outward current is not unique to α-cells

**Figure 4.**
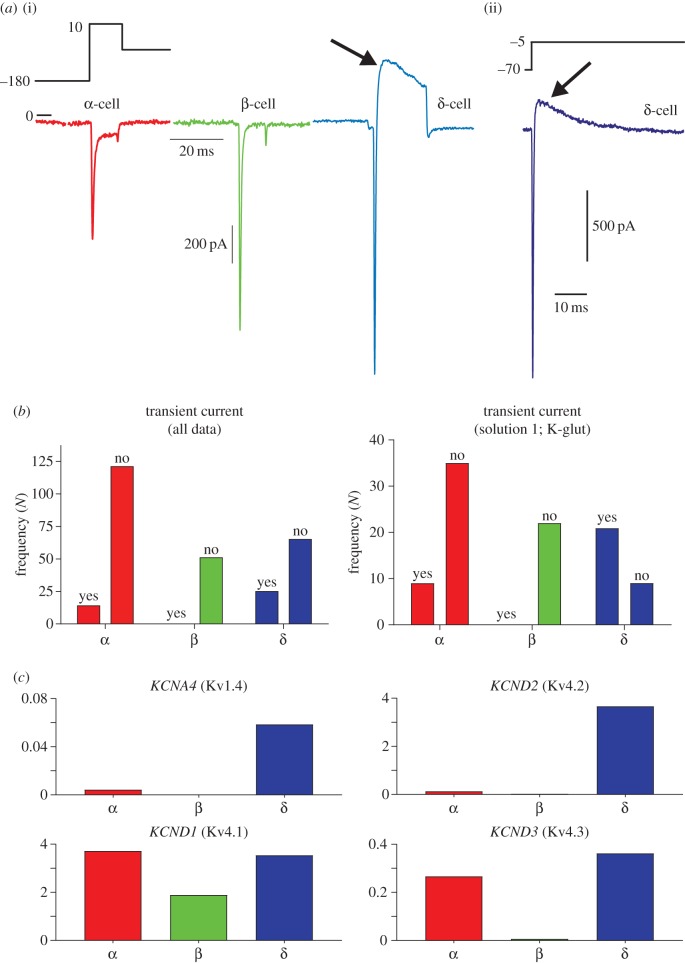
The A-type channel is expressed in both α- and δ-cells. (*a*) The presence of a transient outward current (arrow) was characterized in each of the 288 cell recordings. (*b*) The presence of a transient outward current (A-type) was not a feature unique to α-cells; it was actually most prevalent in δ-cells. *N* = 288 recordings. (*c*) For each cell type, we represented the number of cells that did (yes) and did not (no) exhibit A-type currents in recordings made in intracellular solution 1 (K-glut) only. *N* = 93 recordings. (*d*) The genes encoding TEA-resistant Kv channels of A-type (fast inactivating) are differentially expressed in both α-cells and δ-cells. Transcriptome data from DiGruccio *et al.* [59], reproduced with permission. (Online version in colour.)

Many groups have used the presence of a transient TEA-resistant outward current (putatively an A-type K^+^ current) to define an α-cell [29,31,38,46–48]. We therefore characterized the presence of this current in all recordings (figure 4). Transient outward currents were seen in 14/141 (10%) α-cells, 0/56 (0%) β-cells and 23/91 (25%) δ-cells (figure 4*b*). The presence of a transient outward current was statistically different between δ-cells and α-cells (*p* = 0.0029). When only recordings with intracellular solution 1 (K-glut) were considered, its prevalence in δ-cells (67%) was also greater than that in α-cells (20%; *p* = 0.0001; figure 4*b*).

### 3.7. A binary logistic regression model for identifying β-cells versus non-β-cells

**Figure 5.**
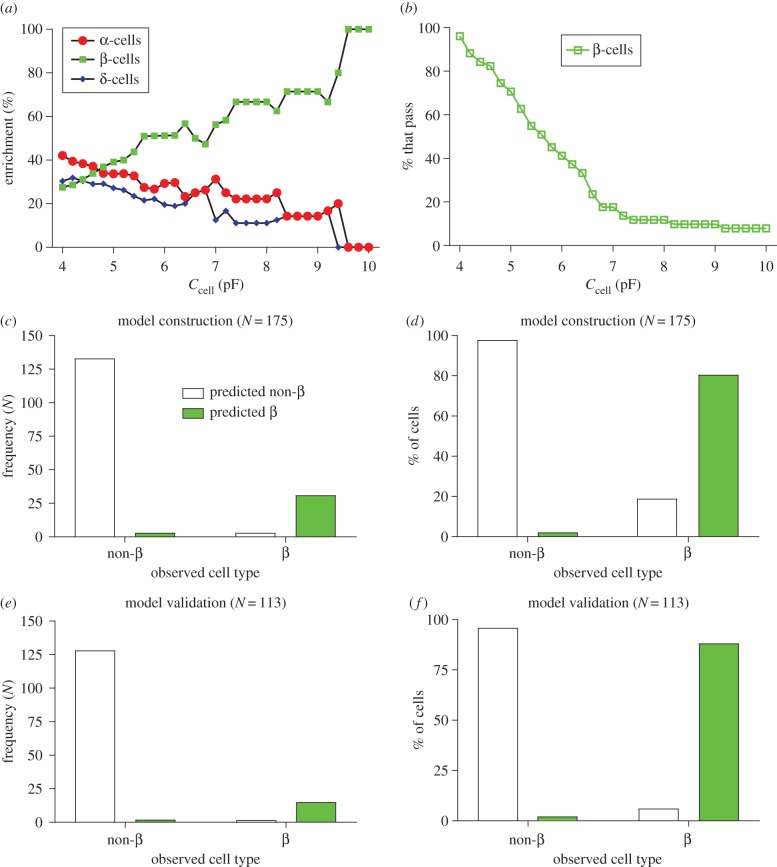
A binary logistic regression model is able to reliable distinguish β- from non-β-cells. We attempted to distinguish β-cells from non-β-cells using a simple criterion. We considered β-cells as having a *C*_cell_ > a fixed cut-off (4–10 pF in 0.2 pF increments; see dashed lines in figure 1*a*). We calculated, for this cut-off, (*a*) the percentage of cells that pass this criterion that are β-cells (β-cell enrichment) and (*b*) the percentage of β-cells from the total β-cell population that pass this criterion (% that pass). Increasing the cut-off to 9.4 pF enriched the β-cell population to 100%, but significantly reduced the number of β-cells passing the criterion (to 4/56 cells (7%)). (*c*,*d*) A binary logistic regression model for distinguishing β- from non-β-cells. The model could accurately distinguish cells in the model construction dataset. 97.8% of non-β-cells were assigned as non-β-cells by the model, and 91.2% of β-cells were assigned as β-cells by the model. (*e*,*f*) The model could also accurately distinguish β-cells from non-β-cells in the model validation dataset. (Online version in colour.)

Electrophysiological criteria have been employed in many studies to distinguish β-cells from non-β-cells. For example, islet cells with *C*_cell_ > 5pF [42] and *C*_cell_ > 6pF [13] have been considered to be β-cells. We therefore investigated whether a simple rule based on *C*_cell_ could distinguish β-cells from non-β-cells. The number of cells with *C*_cell_ > a fixed cut-off (4–10 pF in 0.2 pF increments) were counted. The percentage of cells that passed the criterion that were β-cells (β-cell enrichment; figure 5*a*) and the percentage of all β-cells that pass this criterion (figure 5*b*) were then calculated. For example, 41 cells passed the criterion *C*_cell_ > 6 pF; 12 α-cells, 21 β-cells and eight δ-cells. Therefore, this rule only enriched β-cells in the sample to 51%. Moreover, 35 (56−21) β-cells did not pass this criterion; a 63% reduction in potential sample size. The results were still poor when we applied a stricter criterion; only four β-cells passed the criterion *C*_cell_ > 9.4 pF (100% enrichment), but this came with a 92% reduction in sample size (4/56 β-cells). We conclude that using *C*_cell_ alone to distinguish β-cells from non-β-cells is inadequate.

We therefore used the model construction dataset to construct a binary logistic regression model, to determine whether the electrophysiological variables could collectively distinguish β-cells from non-β-cells (figure 5*c*–*f*). The electrophysiological variables significantly predicting cell type (β-cells from non-β-cells) included *C*_cell_ and *I*_max_. The model was able to distinguish β-cells from non-β-cells with 91% accuracy in the model construction dataset; 32/40 β-cells were correctly assigned as β-cells, and 127/135 non-β-cells were assigned as non-β-cells (figure 5*c*,*d*). When the model was applied to the model validation dataset it again could identify β-cells from non-β-cells with 97% accuracy (figure 5*e*,*f*). We conclude that, when taken together, the electrophysiological variables quantified can distinguish β-cells from non-β-cells with a high degree of accuracy.

### 3.8. A multinomial logistic regression model for identifying cell type

**Figure 6.**
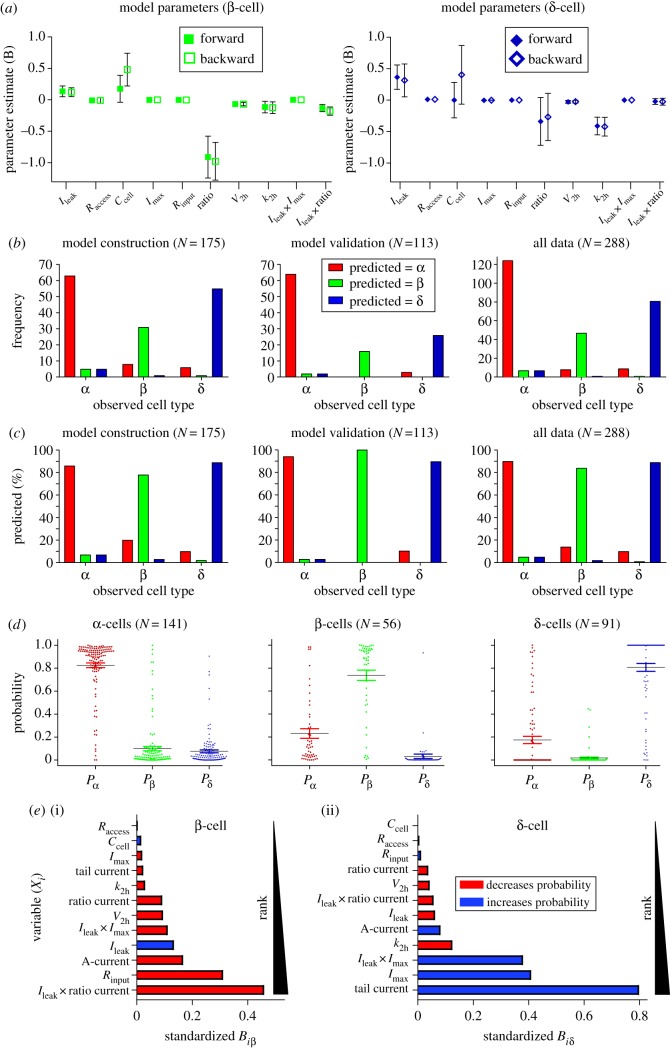
A multinomial logistic regression model is able to reliably identify islet cell type. A multinomial logistic regression model was constructed using the model construction dataset (*N* = 175 cell recordings). (*a*) The forward-entry and backward-elimination methods of variable selection resulted in a final model with similar parameter values. (*b*) The forward-entry model was used to predict islet cell type in the model construction, model validation and entire dataset. Bar chart of observed (by immunocytochemistry) cell type by cell type predicted by the model. (*c*) Percentage of cells correctly identified by the model, for each cell type, in each dataset. *N* = frequency (cell count). (*d*) For all 288 cells, the probability (according to the model) that the cell is an α- (*P*_α_), β- (*P*_β_) and δ-cell (*P*_δ_) was calculated. The maximum of these was used to determine the cell type as identified by the model. (*e*) Standardized model coefficients. The coefficients from the model (equation (B 1), appendix B) were standardized by the method described by Menard [60], so that their importance for determining cell type could be ranked. (*e*(i)) Standardized model coefficients were calculated for the β-cell parameters *B_i_*_β_ (table 4, equation (B 1)). A red (blue, respectively) colour indicates that a SD increase in this variable decreases (increases) the probability that the cell type is β-cell (versus α-cell). (*e*(ii)) A similar ranking was conducted for the variables and δ-cell prediction. (Online version in colour.)

A multinomial logistic regression model was developed to investigate whether the electrophysiological variables could be used together to identify all three cell types, rather than just distinguish β-cells from non-β-cells. The model construction dataset was used for fitting the model parameters (table 3). The modelling process (see appendix B) yielded a final model based on 10 electrophysiological variables (figure 6 and table 4). Importantly, potential confounders, such as animal strain and intracellular solution, did not significantly increase the maximum likelihood of observing the sample values. The model was stable; both forward-entry and backward-elimination methods of variable selection produced a model with similar variables and parameter estimates (figure 6*a* and table 4). The final model constructed with the forward-entry method included the electrophysiological variables *I*_leak_, *R*_access_, *C*_cell_, *k*_h_, *I*_max_, *R*_input_, transient current, ratio current, *V*_2 h_ and tail current. In what follows, this model is used to predict islet cell type.

**Table 3.** Datasets used for model construction and model validation. One-way ANOVA with Kruskal–Wallis post hoc tests; *p*[α versus β], *p*[α versus δ], *p*[β versus δ]. For categorical variables, the count frequency in each category (Y/N = yes/no) is reported and the post hoc *p*-values are computed as described in Sharpe [57]. Transient current: [*N*] = number of recordings in intracellular solution 1 (K^+^-Glut solution). See table 1 for variable descriptions.

variables (*X_i_*)	α-cells			β-cells			δ-cells			*p* [α versus β]	*p* [α versus δ]	*p* [β versus δ]
	mean	s.e.m.	*N*	mean	s.e.m.	*N*	mean	s.e.m.	*N*			
model construction dataset												
*I*_leak_ (pA)	−12.1	1.5	73	−19.3	3.0	40	−20.4	2.6	62	0.077	0.015	0.99
*R*_input_ (GΩ)	2.2	0.2	73	0.9	0.1	40	1.9	0.2	62	<0.001	0.99	<0.001
*C*_cell_ (pF)	4.3	0.2	73	6.0	0.4	40	4.5	0.2	62	<0.001	0.87	<0.001
*R*_access_ (MΩ)	22.7	0.9	73	22.9	1.4	40	22.6	1.2	62	0.99	0.99	0.99
*K*_h_ (mV)	9.6	0.5	73	12.6	1.0	40	7.9	0.5	62	0.022	0.021	<0.001
*I*_max_ (pA)	−492	29	73	−702	59	40	−831	44	62	0.01	<0.001	0.18
*V*_2 h_ (mV)	−39.7	2.1	73	−78.0	3.7	40	−42.4	2.4	62	<0.001	0.43	<0.001
tail current (yes)	0		73	0		40	40/62		62	0.086	<0.001	<0.001
transient current (yes)	11 [24]		73	0 [15]		40	16 [16]		62	0.0318	0.1218	0.0008
double sigmoid (yes)	11		73	32		40	9		62	<0.001	0.8023	<0.0001
model validation dataset												
*I*_leak_ (pA)	−11.4	1.8	68	−115	3.1	16	−22.9	3.5	29	0.42	0.003	0.99
*R*_input_ (*G*Ω)	3.1	0.5	68	1.1	0.2	16	1.6	0.3	29	0.03	0.174	0.68
*C*_cell_ (pF)	4.5	0.1	68	5.3	0.3	16	4.3	0.2	29	0.016	0.99	0.078
*R*_access_ (MΩ)	21.8	0.9	68	23.9	2.0	16	26.5	1.5	29	0.54	0.018	0.54
*k*_h_ (mV)	9.3	0.5	68	14.8	1.4	16	7.2	0.3	29	0.004	0.042	<0.001
*I*_max_ (pA)	−432	22	68	−783	86	16	−876	68	29	0.003	<0.001	0.99
*V*_2 h_ (mV)	−36.9	1.9	68	−79.5	3.5	16	−39.2	2.6	29	<0.001	0.48	<0.001
tail current (yes)	0		68	0		16	19		29	0.055	<0.001	0.007
transient current (yes)	3 [21]		68	0 [7]		16	7 [11]		29	0.99	<0.001	0.008
double sigmoid (yes)	6		68	9		16	0		29	<0.001	0.7059	<0.001

**Table 4.** Parameter estimates for a multinomial logistic regression model for predicting islet cell type. A multinomial logistic regression model of islet cell type, as written in equation (B 1), was constructed using the model construction dataset (*N* = 175 cells). Both forward-entry and backward-elimination approaches were taken to determine which variables to include in the model (*X_i_*) and their associated parameter values (*B_i_*_α_, *B_i_*_β_). The parameter estimates are relative to the α-cell reference category. For variable descriptions, see table 1.

		forward entry			backward elimination		
	variable (*X_i_*)	*B_i_*_β_	s.e.m.	sig.	*B_i_*_β_	s.e.m.	sig.
β-cell variables	intercept	−23.1	2.2	<0.001	−31.6	4400	0.99
	*I*_leak_	0.13	0.09	0.12	0.122	0.07	0.081
	*R*_access_	−8.8 × 10^−3^	43 × 10^−3^	0.84	10 × 10^−3^	47 × 10^−3^	0.824
	*C*_cell_	0.18	0.21	0.41	0.48	0.26	0.064
	*I*_max_	−10 × 10^−3^	17 × 10^−3^	0.553	0	0.002	0.97
	*R*_input_	−7.4 × 10^−4^	3.6 × 10^−4^	0.04	−1 × 10^−3^	0	0.022
	ratio current	−0.91	0.63	0.151	−0.98	0.69	0.16
	transient current (Y/N)	22.4	0	<0.001	24.9	0	<0.001
	*V*_2 h_	−6.8 × 10^−2^	2.7 × 10^−2^	0.009	−6.8 × 10^−2^	2.6 × 10^−2^	0.009
	tail current (Y/N)	−0.99	0	0.99	4.53	4400	0.99
	*k*_2 h_	−0.11	0.09	0.21	−0.13	0.09	0.18
	*I*_leak_ × *I*_max_	−11 × 10^−6^	13 × 10^−6^	0.39	−22 × 10^−6^	14 × 10^−5^	0.87
	*I*_leak_ × ratio current	−0.13	0.06	0.02	−0.18	0.07	0.007

	variable (*X_i_*)	*B_i_*_δ_	s.e.m.	sig.	*B_i_*_δ_	s.e.m.	sig.
δ-cell variables	intercept	31.9	2535	0.990	31.4	3137	0.992
	*I*_leak_	0.37	0.194	0.060	0.314	0.261	0.229
	*R*_access_	0.012	0.043	0.784	0.011	0.045	0.803
	*C*_cell_	0.001	0.282	0.996	0.402	0.467	0.390
	*I*_max_	−0.003	0.002	0.111	−0.002	0.002	0.342
	*R*_input_	0.000	0.000	0.443	0.000	0.000	0.439
	ratio current	−0.34	0.38	0.373	−0.267	0.375	0.475
	transient current (Y/N)	−3.6	0.92	0.000	−3.713	0.981	0.000
	*V*_2 h_	−0.028	0.024	0.239	−0.025	0.023	0.279
	tail current (Y/N)	−29.6	2535	0.991	−30.1	3137	0.992
	*k*_2 h_	−0.411	0.14	0.004	−0.422	0.15	0.005
	*I*_leak_ × *I*_max_	0.000	0.000	0.069	0.000	0.000	0.056
	*I*_leak_ × ratio current	−0.021	0.05	0.64	−0.025	0.055	0.641

The model was applied to the model validation dataset (*N* = 113; table 3) to see how well it can identify cell type. The model identified α-cells with 94% accuracy, δ-cells with 90% accuracy and β-cells with 100% accuracy (figure 6*b*,*c*). These data demonstrate that the model is applicable to other datasets, as it can predict islet cell type in the model validation dataset with an overall accuracy of 94%.

To rank the variables in the model by their importance for identifying islet cell type, standardized coefficients were calculated as described by Menard [60]. *V*_2 h_ and *C*_cell_—variables typically used to distinguish β-cells from non-β-cells [3,11,29,35,37–41,44–47,50]—ranked low (eighth and 12th, respectively) on the list (figure 6*e*(i)). The most important variable for distinguishing δ-cells from α-cells was the presence of a slow Ca^2+^ tail current (figure 6*e*(ii)). The presence of an A-current—which has frequently been employed to distinguish these two cell types [29,31,38,46–48]—was not the highest ranking variable. The variable that ranked second was *I*_max_, indicating that a large Na^+^ current is an important distinguishing feature of δ-cells from α-cells. These findings do not conform to standard practice for identifying cell type and therefore highlight the importance of using our multinomial logistic regression model to identify cell type.

### 3.9. Incorrectly identified α-cells have β-cell-like characteristics

**Figure 7.**
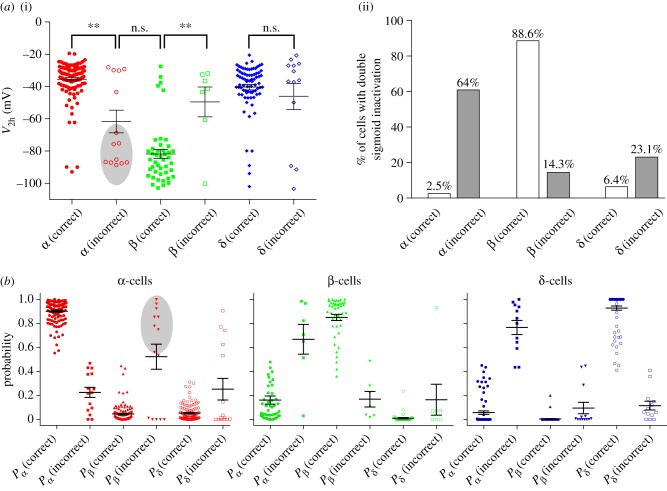
Properties of cells incorrectly identified by the multinomial logistic regression model. Cells were divided into those correctly and incorrectly identified by the model. (*a*(i)) α-cells that were incorrectly identified had a significantly hyperpolarized *V*_2 h_ compared with α-cells that were correctly predicted by the model. Furthermore, the incorrectly identified α-cells had a *V*_2 h_ that did not differ from (correctly identified) β-cells. (*a*(ii)) The proportion of cells exhibiting a double sigmoid was different across cells correctly and incorrectly identified by the model. (*b*) Model probabilities for each cell type, split by those correctly and incorrectly identified by the model. The shaded region pertains to the α-cells with hyperpolarized *V*_2 h_ (cells in shaded region in *a*(i)). One-way ANOVA; n.s., not significant, *p < 0.05, **p < 0.01. (Online version in colour.)

We characterized the cells whose cell type was incorrectly identified by the model (figure 7). The model incorrectly identified 14/141 α-cells. In those α-cells incorrectly assigned (as β- or δ-cells), the measured *V*_2 h_ was significantly hyperpolarized (−64.1 ± 7.1) compared with correctly assigned α-cells (−35.3 ± 1.1; *p* = 0.01). Moreover, it did not differ from that of β-cells that were correctly identified by the model (−81.8 ± 2.8; *p* = 0.42; figure 7*a*(i)). Furthermore, 64% of the incorrectly identified α-cells (9/14) exhibited a double sigmoidal *h*_∞_, compared with 2.5% of cells in the correctly labelled α-cell population (figure 7*a*(ii)). These incorrectly identified α-cells therefore have ‘β-cell-like’ Na^+^ channel properties. The model supported this idea; the probability that the incorrectly identified α-cells were β-cells (*P*_β_ = 0.52 ± 0.1) was significantly larger than for correctly identified α-cells (*P*_β_ = 0.05 ± 0.01; *p* = 0.001; figure 7*b*). In particular, the model revealed that these α-cells have ‘β-cell-like’ properties.

### 3.10. The model can identify islet cell type in mice with a hyperglycaemic phenotype

**Figure 8.**
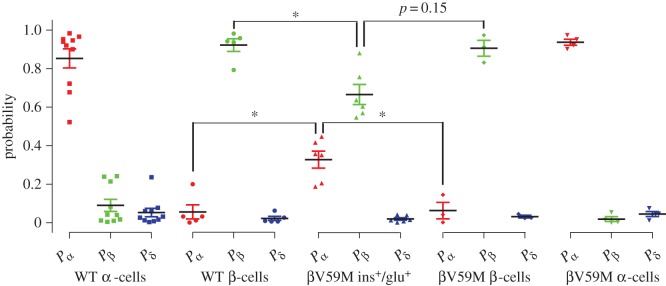
The multinomial logistic regression model can predict cell type in an animal strain of diabetes. Cell type was predicted for cells recorded from an animal with a hyperglycaemic phenotype (βV59M) and wild-type controls (WT). The model outputted the probability that each cell is an α-cell (*P*_α_), β-cell (*P*_β_) or δ-cell (*P*_δ_), from which the cell type predicted by the model could be determined. Cells that stained double positive for insulin and glucagon (ins^+^/glu^+^) had a larger *P*_α_ and smaller *P*_β_ than β-cells from WT and βV59M, suggesting that these cells had both α- and β-cell electrophysiological properties. One-way ANOVA, *p < 0.05. (Online version in colour.)

To investigate whether the model could accurately identify islet cell type from mice with a hyperglycaemic phenotype, an additional 13 cell recordings were made in βV59M mice and 15 in wild-type control mice (WT; figure 8). The model identified cell type in recordings from WT islets with 100% accuracy, identifying all five β-cells (*P*_β_ = 0.95 ± 0.04) and 10 α-cells (*P*_α_ = 0.86 ± 0.05) correctly. In βV59M mice, the model correctly identified all three β-cells (*P*_β_ = 0.91 ± 0.04) and four α-cells (*P*_α_ = 0.94 ± 0.02). The remaining six recordings from βV59M mice were revealed (by immunocytochemical staining) to be from cells co-expressing insulin and glucagon (ins^+^/glu^+^). The model identified all of these cells as β-cells (*P*_β_ = 0.67 ± 0.05). The probability that these cells were α-cells (*P*_α_ = 0.37 ± 0.05), as predicted by the model, was significantly larger than the probability that β-cells from WT (*P*_α_ = 0.06 ± 0.04; *p* = 0.022) or βV59M (*P*_α_ = 0.07 ± 0.04; *p* = 0.15) mice were identified as α-cells by the model. Furthermore, the model was less certain that these βV59M ins^+^/glu^+^ cells were β-cells; *P*_β_ in βV59M ins^+^/glu^+^ cells was smaller than in WT (*p* = 0.019) and βV59M (*p* = 0.04) β-cells. Therefore, although the model predicted these six βV59M ins^+^/glu^+^ cells to be β-cells, it also revealed that they had ‘α-cell-like’ electrophysiological properties.

## 4. Discussion

Here we have quantified numerous electrophysiological variables in α-, β- and δ-cells from intact mouse islets. Our study highlights the perils of using a single electrophysiological variable to distinguish cell type and demonstrates that some established methods for functional identifying cell type are misleading (figures 1–4). We show, by constructing a multinomial logistic regression model (figure 6), that multiple electrophysiological variables can be used to predict islet cell type with 94% accuracy. The mathematical model was also able to identify cells from a diabetic mouse, and could distinguish cells in this mouse that were positive for both insulin and glucagon (figure 8).

### 4.1. Functionally identifying islet cell type based on a few electrophysiological properties

When recording membrane potential in the perforated patch-clamp configuration, the electrical activity of the cell in response to application of glucose is one method by which cell identity can be alluded to. The aim of this study was to provide a tool for accurately identifying cell type when membrane potential recording is not required or cannot be used (namely in voltage-clamp experiments under the standard whole-cell configuration).

Under the standard whole-cell configuration, α-, β- and δ-cells in mouse islets are known to exhibit electrophysiological properties that differ. These properties are often used to functionally identify the cell type [11,13,29,31,34–36,39–42,44,46–48,50,61]. For example, an electrophysiological feature that is commonly employed to distinguish cell type is the cell capacitance [3,13,35–41,43,44], which is largest in β-cells. Some studies have employed a criterion based on cell capacitance to distinguish β-cells from non-β-cells [13,34,42]. However, we show that functionally identifying β-cells from non-β-cells using cell capacitance is unreliable (figure 5*a*,*b*). Criteria based on cell capacitance alone may moderately enrich the cell type of interest, but will significantly reduce the sample size. Thus, even if subsequent criteria are applied (e.g. pertaining to Na^+^ current properties; see Rolland *et al.* [42]), the dataset will already be considerably reduced in size and not representative of the population. Furthermore, although our large dataset demonstrated many differences in electrophysiological properties across cell type, no single feature was able to distinguish islet cell type (table 2). A better method of identifying islet cell type is therefore required.

### 4.2. A multinomial logistic regression model for identifying islet cell type

To determine whether the electrophysiological features we measured could, collectively, be used to predict islet cell type, we constructed a multinomial logistic regression model. This model was able to predict islet cell type with 94% accuracy (figure 6). It requires only a few standard electrophysiological variables as input. Its accuracy and speed could aid online identification of cell type and can replace the lengthy immunocytochemical and imaging procedures. This model demonstrated that Na^+^ current variables, the input resistance (1/*G*) and cell capacitance are significant predictors of cell type, when important experimental confounders (e.g. access resistance and leak current) are controlled for. Interestingly, the model revealed that the leak current—an experimental confounder—is a significant predictor of cell type (table 4). It is therefore important to consider such experimental confounders when using electrophysiological variables to identify cell type.

For each recorded cell, the model generated probabilities *P*_α_, *P*_β_ and *P*_δ_—the maximum of which yielded the cell type predicted by the model. The model could correctly identify cell type in mice with a diabetic phenotype [54] and identify cells that were positive for both insulin and glucagon. It may therefore help to understand the electrophysiological properties of cells undergoing reprogramming [62].

### 4.3. A-type K^+^ current (transient outward current) as an identifier of cell type

The presence/absence of an A-current has been used in many studies as an identifying feature for α-cells/δ-cells, respectively [29,31,38,46–48]. Our analysis of a large sample of cells revealed that the notion that the A-current is an identifying feature of α-cells is false (figure 4). We demonstrate that it is a feature of both δ-cells (67% of δ-cells exhibited a transient outward current) and α-cells (20%; figure 4*c*). This is supported by transcriptome data from DiGruccio *et al.* [59] that report expression of genes encoding A-type K^+^ channels in both α- and δ-cells (figure 4*d*) [59]. *KCNA4* and *KCND2* are preferentially expressed in δ-cells, and *KCND1* and *KCND3* exhibit similar levels of expression in α- and δ-cells. Similarly, Adriaenssens *et al.* [56] recently reported genes differentially expressed between α-, β- and δ-cells; genes encoded by A-type channels were not found to exhibit significant expression changes between α- and δ-cells [56]. In conclusion, the presence of an A-type current is not unique to α-cells, and should therefore be avoided as an identifier of cell type.

How do we reconcile this fact with the observation that 4-aminopyridine (4-AP) reduces glucagon secretion in mouse islets [49]? First, although 4-AP is traditionally seen as a blocker of A-type K^+^ channels [63], it is not selective for K^+^ channels that inactivate; it blocks both slowly inactivating and non-inactivating K^+^ currents of delayed rectifier type, including *Shaker* family members Kv1.1 [64], Kv1.2 [65], Kv1.3 [66], Kv1.5 [67,68] and Kv1.6 [69], as well as *Shab*-related Kv2.1 and *Shaw*-related Kv3.1 [70]. Secondly, if, as our analysis suggests, the A-type current is actually a fingerprint of δ-cells, then blockade of this current will increase action potential width in δ-cells, facilitating somatostatin release. This may decrease glucagon secretion via paracrine inhibition of α-cells [71].

### 4.4. Improved conductance-based models of α-cells

**Figure 9.**
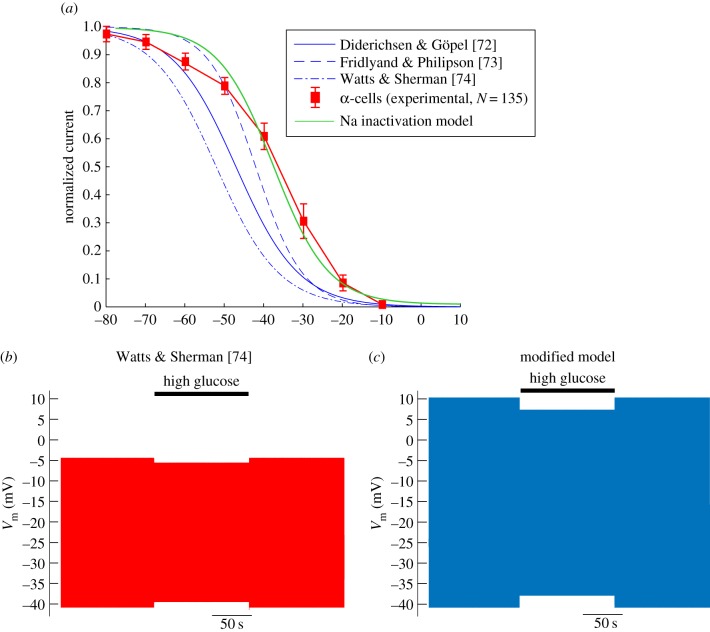
Modifying a conductance-based model of an α-cell improves the fit to experimental data. (*a*) Steady-state Na^+^ current inactivation in three conductance-based models of α-cells [72–74], *N* = 141 experimentally recorded α-cells, and our modified model (Na^+^ inactivation model). Our modified model can be seen to fit the experimental data well. We simulated the model of (*b*) Watts & Sherman [74] and (*c*) our modified model under conditions of high glucose (decreased *G*_KATP_; black line). Our model produced a 6.1 mV change in action potential height, as seen experimentally [11]. In comparison, the model of Watts & Sherman [74] produced moderate changes in action potential height (2.5 mV) and doublet spikes in response to high glucose. (Online version in colour.)

**Figure 10.**
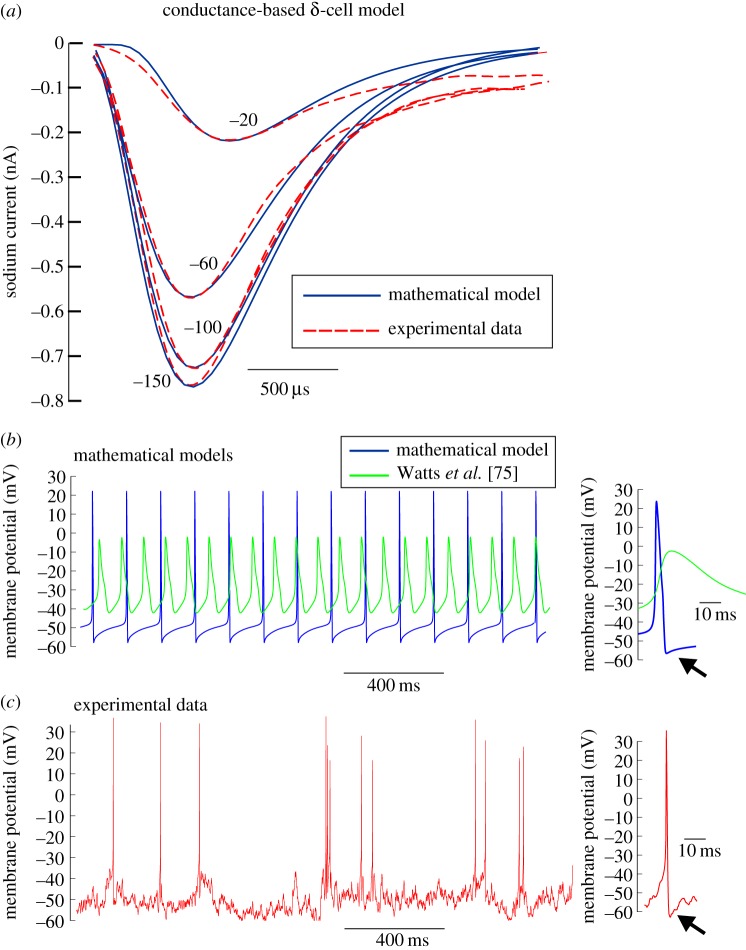
Modifying a conductance-based model of a δ-cell improves the fit to experimental data. We used our large dataset to improve the fit of a conductance-based model of a δ-cell to the experimental data. The δ-cell model of Watts *et al.* [75] was used as the starting model. (*a*) The model was simulated under a Na^+^ current inactivation voltage-step protocol (*V*_cond_ = −180 to 20 mV followed by a test pulse to 0 mV), and the evoked Na^+^ current in the model (solid) was compared with the inward current experimentally recorded from a δ-cell under the same protocol (dashed). The Na^+^ current model parameters were then optimized by the method of Willms *et al.* [76], so that the model fitted the experimental data. The currents shown are in response to *V*_cond_ = −150, −100, −60 and −20 mV. (*b*) Spiking behaviour generated by the model of Watts *et al.* [75] with the reparametrized Na^+^ current model (as shown in *a*) and *C*_cell_ = 4 pF was compared with the original, unaltered δ-cell model by Watts *et al.* [75]. Note that action potentials evoked in the improved model overshoot 0 mV, have short duration and also have a pronounced after-hyperpolarization (arrows; owing to the A-type K^+^ current), as seen in the experimental action potentials recorded from a δ-cell under the perforated patch-clamp configuration (*c*). (Online version in colour.)

To demonstrate the importance of our improved characterization of the electrophysiological properties of α- and δ-cells, we used our findings to develop models of the electrical activity in these cell types (figures 9 and 10).

### 4.5. An improved conductance-based model of α-cell electrical activity

Conductance-based mathematical models of the electrical activity of α-cells have provided us with invaluable insights into the mechanisms regulating glucagon secretion [72–75]. However, parameters used in these models were based on presumptive α-cells identified by traditional electrophysiological criteria [3,29,31,35,38,46], which we have shown here to be inaccurate. The parameters used in these models were therefore not always correct. The recent model of Watts & Sherman [74] includes an A-type K^+^ current which we demonstrate is present in 20% of α-cells (figure 4). It also included Na^+^ current parameters that did not resemble our experimental dataset (figure 9*a*). Furthermore, the cell capacitance reported in previously published models was 5 pF [72–74], which does not resemble *C*_cell_ for α-cells reported here (4.2 ± 0.1 pF; figure 1*a*) or previously [11,45]. These discrepancies may explain why the model of Watts & Sherman [74] produced a small decrease in spike height (2.45 mV) and doublet spikes during simulation of high-glucose conditions (figure 9*b*,*c*), a feature not seen experimentally [11]. We therefore modified this model in the light of our findings (appendix C). When we removed the A-current, decreased *C*_cell_ to 4 pF and modified the Na^+^ current parameters in the model to fit our experimental data, the similarity between the model and the experimental data under simulated high-glucose improved. In particular, in low-glucose conditions, the spike height of the model action potential overshot 0 mV and had an amplitude of more than 50 mV, as seen experimentally [11,52]. Moreover, in high-glucose conditions, the decrease in spike height was larger (6.1 mV), as observed experimentally [11]. These results do not disagree with the results produced from simulations of conductance-based models of α-cells by Watts & Sherman [74]. In fact, we used the model by Watts & Sherman [74] as a starting model (as opposed to the other available models of α-cells) because it correctly captures the phenomenological behaviour seen in the experimental data when high glucose is added. Our improvement of this model, based on our experimental findings, illustrates the importance of using reliable techniques for identifying cell type.

### 4.6. An improved conductance-based model of δ-cell electrical activity

A conductance-based model of δ-cell electrical activity calibrated against experimental data does not exist. Recently, Watts *et al.* [75] generated a conductance-based model of a δ-cell for studying the dynamical interactions between cell types, but this was a modified version of an α-cell model [75]. We therefore developed a model of δ-cell electrical activity, constrained to our experimental data for δ-cells (figure 10 and appendix C). The Na^+^ current kinetics in the model were fitted to experimental data from a δ-cell recording by the improved parameter estimation method proposed by Willms *et al.* [76]. Given that our data demonstrate that δ-cells have slow Ca^2+^ tail currents (figure 3) and A-currents (figure 4), T-type Ca^2+^ and A-type K^+^ currents were included in the model. These modifications produced a good fit between the model and experimental data in response to the Na^+^ inactivation protocol (from a single recording of a δ-cell; figure 10*a*). We also changed *C*_cell_ in the model (from 5 to 4 pF) to fit the experimental data for δ-cells (4.3 ± 0.1 pF, *N* = 91; figure 1*a*). When the model was simulated under current clamp conditions, it produced large-amplitude spikes that overshot 0 mV and had large after-hyperpolarizations (figure 10*b*). Similar spikes were seen experimentally using the perforated patch-clamp configuration (figure 10*c*).

### 4.7. Future directions and conclusions

We have focused our model on characterizing islet cell type from recordings made from intact islets. Some studies, however, use dispersed islet cells. Our model was not tested against recordings from dispersed cells for two reasons. First, cell identification by immunocytochemistry is straightforward in dispersed cells. Second, there is evidence that both cell size and Na^+^ current density are altered in dispersed islet cells [77].

α-, β- and δ-cells in human islets possess very distinct electrophysiological features compared with their mouse counterparts [78–80]. Unlike the mouse, no functional identification exists for human cell type; patch-clamp recordings from human islets are rare. Therefore, identification of cell type demands successful immunocytochemical staining. The difficulties faced when studying the electrophysiological properties of human islet cells are reflected by the sample sizes (typically <10 [78–80]). Furthermore, human islet function is very heterogeneous [11,81,82]. These obstacles have undoubtedly contributed to the slow progress in our understanding of the electrophysiological properties of each cell type in human islets, and how these properties correlate to the phenotype (e.g. non-diabetic/diabetic) of the donor.

The modelling process outlined in this study would also be helpful in the study of human islets. Such a model could be used to determine the key electrophysiological variables that identify cell type, making comprehensible the defining electrophysiological properties of these heterogeneous cells. It could also be used to predict the disease state (non-diabetic/diabetic) of a donor given a set of electrophysiological variables. Such a modelling procedure would illuminate which electrophysiological properties differ in diabetes, while correctly controlling for experimental confounders.

In conclusion, we have conducted a comprehensive analysis of the electrophysiological properties of islet cells traditionally used for identifying cell type, in a large population of recordings. We used this dataset to reveal which electrophysiological fingerprints were reliable for identifying cell type, and then constructed a logistic regression model that can be used to predict islet cell type with 94% accuracy. These data were successfully used to not only predict cell type in diabetic mouse models, but also improve conductance-based models of α- and δ-cells.

## Appendix A: electrophysiological variables quantified

Here we describe the experimental electrophysiological variables quantified from every recorded cell.

**A.1. Whole-cell conductance (*G*)**

The reciprocal of the input resistance (1/*R*_input_), taken from the parameter window of the EPC-10, was computed to yield the whole-cell conductance (*G*). This to a large extent reflects the *K*_ATP_-channel conductance (*G*_KATP_).

**A.2. Na^+^ current variables**

Voltage-gated Na^+^ currents (*I*_Na_) exhibit distinct properties in β- and non-β-cells; these are frequently used to functionally identify cell type [3,11,29,35,37–41,44–47,50]. We therefore sought to characterize these fingerprints in each cell recorded. Steady-state properties of *I*_Na_ were investigated by applying a 200 ms conditioning potential (*V*_cond_ = −180 to 20 mV, 10 mV increments) followed by a 10 ms test pulse to 0 mV. Maximum Na^+^ current amplitude (*I*_max_) was taken as the peak current evoked following a conditioning pulse of *V*_cond_ = −180 mV. For each conditioning potential, the peak current evoked during the test pulse (*I*) was normalized by *I*_max_. This yielded a sigmoid relationship, which represents the steady-state inactivation of the Na^+^ current (*h*_∞_ = *I*/*I*_max_) as a function of *V*_cond_. The data were then fitted with a single sigmoid,

in Matlab v. 6.1 (2000; The MathWorks, Natick, MA). The fit process yielded two biological parameters for inactivation: the half-inactivation (*V*_2 h_) and the slope factor (*k*_h_). It also produced a goodness-of-fit (*R*^2^). Zhang *et al.* [45] recently demonstrated that *h*_∞_ in β-cells exhibits a biphasic shape and fits well with a double sigmoid,

We therefore fitted all data with both a double and single sigmoid function. The fit with the largest *R*^2^ was taken as the most appropriate fit for the *h*_∞_(*V*_cond_) data. If the fit was a double sigmoid, then the value of *V*_2 h_ used to compare with other cell types was the most negative value out of *V*_2 h,1_ and *V*_2 h,2_.

**A.3. Cell capacitance, *R*_access_ and *I*_leak_**

α-, β- and δ-cells differ in cell size and this is frequently used as an identifying feature for cell type [3,13,35–41,43,44]. A proxy for cell size (measurable during whole-cell patch-clamp recordings) is cell capacitance (*C*_cell_). This is taken as the slow component of the capacitive transient, as, after fast capacitance compensation for electrode capacitance, all remaining capacitive transients come from the cell capacitance [83].

The access (series) resistance (*R*_access_) was also recorded as this is a potential confounder of the recorded electrophysiological properties of the cell [84,85]. Similarly, the leak current (*I*_leak_) was recorded.

**A.4. Transient outward and slow tail currents**

In some recordings, application of a conditioning potential *V*_cond_ < −70 mV, followed by a test pulse to 0 mV, evoked a transient outward current that persisted in the presence of 20 mM TEA-Cl. This current, carried by K^+^ and putatively of A-type, has been used to identify α-cells [29,31,38,46–48]. In each cell, we characterized whether such a current was present or absent (transient current = yes/no; table 1).

A current–voltage protocol was also applied to each cell recorded, to determine the peak currents elicited by voltage steps to different membrane potentials. Voltage steps of amplitude −100 to 30 mV (10 mV increments) were applied from a holding potential of −70 mV, and the evoked current recorded. This peak current was recorded (*I*_max70_). In some cells, a slowly deactivating inward tail current (slow tail current) was observed on termination of the voltage step to 30 mV. This slow tail current, presumed to be a T-type Ca^2+^ current, has been used to identify islet cell type [29,31,38,46]. In each recorded cell, we characterized whether this slow tail current was present or absent (tail current = yes/no; table 1). As this characteristic is descriptive and subjective, we fitted a single exponential to the decay time course of this slow tail current; if the time constant of decay was more than 1.5 ms then a slow tail current was considered to be present (tail current = yes).

## Appendix B: multinomial logistic regression model for identifying islet cell type

**B.1. Multinomial logistic regression analysis**

Our aim was to use the experimental variables calculated (table 1) to construct a regression model for predicting cell type (α, β or δ). Because the dependent variable is categorical with more than two levels, we fitted a multinomial logistic regression model to the experimental data. The benefit of this form of regression model is that it allows independent variables to be both categorical and continuous. It also accounts for experimental confounders [86] and how these influence identification of cell type. The model has the form

B 1

Here, *B_i_*_α_ and *B_i_*_β_ are 2(*n* + 1) parameters determined by the modelling fitting process and *X_i_* are the *n* independent variables (identifying features). For example, our model may include *X*_1_ = *V*_2 h_ and *X*_2_ = *C*_cell_. Given a cell and its set of values of *X_i_*, the model produces *P*_α_ and *P*_β_—the probabilities that the cell is an α-cell and β-cell, respectively. This model also yields the probability that a cell is a δ-cell; *P_δ_* = 1 − *P_α_* − *P_β_*. What remains to be outlined is how (i) *B_i_*_α_ and *B_i_*_β_ are determined and (ii) the variables *X_i_* are chosen.

**B.2. Model construction**

In the model fitting process, parameter values (*B_i_*_α_, *B_i_*_β_) were chosen so that they maximized the likelihood of observing the sampled values *X_i_* [87]. For the model fitting process, *N* = 175 recordings made in mice with a normoglycaemic phenotype (60% of the normoglycaemic dataset) were used as the sample values, and the model was fitted to these samples (table 3). This dataset is referred to as the ‘model construction’ dataset. All logistic regression models presented were constructed using this dataset.

**B.3. Models with one independent variable**

To understand whether a particular independent variable (e.g. *X*_1_ = *V*_2 h_) can alone identify cell type, equation (B 1) was fitted to the experimental data with just this single independent variable. This model takes the form

B 2

where *B*_0α_, *B*_1α_, *B*_0β_ and *B*_1β_ are parameters determined by the fitting process and *X*_1_ is the independent variable of interest. This model can be used to understand how reliable *X*_1_ alone is at correctly identifying cell type.

**B.4. Model with more than one independent variable—the variable selection process**

To understand whether the electrophysiological variables could be used together to accurately predict cell type, a forward-entry approach was taken. A variable was entered into the model as a new independent variable *X_i_* if it significantly increased the maximum likelihood of observing the sampled values [87]. The variables considered for this process are precisely those described above (table 1). A backward-elimination method was also applied to test stability of the variable selection process.

Certain variables were forced to be in the model. Experimental confounders were accounted for by inclusion in the initial model [86]. Because *R*_access_ can influence the observed current–voltage relationship and the temporal resolution of recorded currents [84,85], this variable was considered as an experimental confounder and forced to be in the model. For similar reasons, multiplicative variables were included in the variable selection process; *R*_access_ · *I*_max_, *R*_access_ · *V*_2 h_ and *R*_access_ · *k*_h_ were subjected to maximum-likelihood criteria for inclusion in the model. *I*_leak_, which is a measure of the seal quality, was forced to be in the model. The strain of the animal from which the cell recording was taken was considered as experimental confounders (influencing cell type). This categorical variable was not forced to be in the model but instead subjected to the aforementioned maximum-likelihood criteria. Many studies report *I*_max_ normalized to *C*_cell_ because this can bias the current amplitude; the larger the cell area, the larger the current recorded. We could account for this in the model by including the variable *C*_cell_ · *I*_max_ in the modelling process and seeing whether this explained any variance in cell type. Two intracellular solutions were used for recording (solutions 1 and 2). As the solution used may influence the magnitude of the outward currents observable, and outward (namely A type) currents are used to identify cell type, we included the variable intracellular solution (solution 1/solution 2) as a confounder in the model.

The final model from this process was used to calculate the probability that any given cell (with sample values *X_i_* for *i* = 0, … ,*n*) is an α-cell (*P*_α_), β-cell (*P*_β_) or δ-cell (*P*_δ_). The maximum of these three computed probabilities determines the cell type predicted by the model and can be compared with the observed cell type (confirmed by immunocytochemistry).

**B.5. Model validation**

Following the model construction process, the model was validated. To ensure that the model fit is generalizable to other datasets, a second dataset (distinct from the model construction dataset) was used. This ‘model validation’ dataset consisted of the remaining *N* = 113 cell recordings made from mice with a normoglycaemic phenotype (40% of the normoglycaemic dataset; table 3). For each cell recording (with experimental variables *X_i_*), the values *X_i_* were entered into equation (B 1) and the probabilities (*P*_α_, *P*_β_, *P*_δ_) computed. The cell type predicted by the model could then be compared with the observed cell type.

## Appendix C: conductance-based models of electrical activity

**C.1. Conductance-based models of α-cells**

We studied how any discrepancies in the identifying features of α-cells would change the behaviour of previously published conductance-based models of α-cells (which have relied on these identifying features to constrain model parameters). To do this, we used the recent model by Watts & Sherman [74]. The unmodified model is

where *C*_cell_ is the cell capacitance; *I*_CaL_, *I*_CaN_ and *I*_CaT_ are L-, N- and T-type voltage-dependent Ca^2+^ currents, respectively; *I*_Na_ is a voltage-dependent Na^+^ current; *I*_K_ is a delayed rectifier K^+^ current; *I*_KA_ is an A-type voltage-dependent K^+^ current; *I*_K(ATP)_ is an ATP-sensitive K^+^ current; *I*_L_ is a leak current; and *I*_SOC_ is a store-operated Ca^2+^ current. A full description of this model can be found in Watts & Sherman [74] and the model code can be obtained from GitHub (https://github.com/IsletCellType/IsletCellType_GitHub).

**C.2. Conductance-based models of δ-cells**

Similarly, we studied how any discrepancies in the identifying features of δ-cells would change the behaviour of previously published conductance-based models of δ-cells (which have relied on these identifying features to constrain model parameters). To do this, we used the recent model by Watts *et al.* [75]. The unmodified model is

where *C*_cell_ is the cell capacitance; *I*_CaL_ and *I*_CaN_ are the L- and N-type voltage-dependent Ca^2+^ currents, respectively; *I*_Na_ is a voltage-dependent Na^+^ current; *I*_K_ is a delayed rectifier K^+^ current; *I*_KA_ is an A-type voltage-dependent K^+^ current; *I*_K(ATP)_ is an ATP-sensitive K^+^ current; and *I*_L_ is a leak current. The GABA current was excluded from the model as we were not modelling paracrine signalling. The parameter values of *I*_K_, *I*_K(ATP)_, *I*_KA_ and *I*_L_ were left unmodified. The parameter values of *I*_Na_ were fitted to experimental data by the process described by Willms *et al.* [76]. The only further modification to this model was that the time constants of the voltage-gated Ca^2+^ channels were decreased, because action potentials generated by the model were seen to be too broad in comparison with experimental data. This model is available online at GitHub (https://github.com/IsletCellType/IsletCellType_GitHub).
